# Intratumoral Heterogeneity and Immune Microenvironment in Hepatoblastoma Revealed by Single‐Cell RNA Sequencing

**DOI:** 10.1111/jcmm.70482

**Published:** 2025-03-18

**Authors:** Mingdi Ma, Chen Jin, Qian Dong

**Affiliations:** ^1^ Department of Pediatric Surgery The Affiliated Hospital of Qingdao University Qingdao China; ^2^ Shandong Key Laboratory of Digital Medicine and Computer Assisted Surgery The Affiliated Hospital of Qingdao University Qingdao China

**Keywords:** Hepatoblastoma (HB), intra‐tumour heterogeneity, single‐cell RNA‐Seq, tumour microenvironment

## Abstract

Hepatoblastoma (HB) is a common paediatric liver malignancy characterised by significant intratumoral heterogeneity and a complex tumour microenvironment (TME). Using single‐cell RNA sequencing (scRNA‐seq), we analysed 43,592 cells from three tumour regions and adjacent normal tissue of an HB patient. Our study revealed distinct cellular compositions and varying degrees of malignancy across different tumour regions, with the T1 region showing the highest malignancy and overexpression of HMGB2 and TOP2A. Survival analysis demonstrated that high HMGB2 expression is associated with poor prognosis and increased recurrence, suggesting its potential as a prognostic marker. Additionally, we identified a diverse immune microenvironment enriched with regulatory T cells (Tregs) and CD8^+^ effector memory T cells (Tem), indicating potential immune evasion mechanisms. Notably, CTLA‐4 and PD‐1 were highly expressed in Tregs and Tem cells, highlighting their potential as immunotherapy targets. Myeloid cells, including Kupffer cells and dendritic cells, also exhibited distinct functional roles in different tumour regions. This study provides the first comprehensive single‐cell atlas of HB, revealing critical insights into its intratumoral heterogeneity and immune microenvironment. Our findings not only advance the understanding of HB biology but also offer new directions for precision medicine, including the development of targeted therapies and immunotherapeutic strategies to improve patient outcomes.

## 1 Introduction

Hepatoblastoma (HB), derived from embryonic liver parenchyma cells and hepatoblast cells, is the most common primary liver malignant tumour in children, with an incidence rate of 1.3–1.5 ppm [1, 2]. HB primarily affects young children between 2 months and 3 years of age and is associated with preterm birth and birth defects [3]. The 3‐year survival rate for patients with low‐risk HB was 91% [4] while for high‐risk patients, such as those with larger tumour extension (PRETEXT IV), vascular invasion or metastasis, the survival rate drops to 65% [5]. Studies have shown that HB tumours consist of a heterogeneous population of stem/progenitor cells, originating from hepatoblasts that undergo undesired genetic and epigenetic aberrations during development [6, 7, 8]. Although surgery and chemotherapy remain the primary treatment methods for HB, a significant proportion of patients experience recurrence and/or metastasis, leading to poor outcomes. A major obstacle to precision medicine is the lack of understanding of tumour heterogeneity and the underlying molecular mechanisms.

The tumour microenvironment (TME) consists of cancer cells, infiltrating immune cells, stromal cells, and other types of cells, as well as non‐cellular tissue components [9]. TME is as complex and heterogeneous as a cancer cell compartment [10, 11]. Studies have shown that macrophages, T cells, and fibroblasts are highly heterogeneous [12, 13, 14, 15]. Despite advances in our understanding of the TME, the mechanisms by which cellular heterogeneity drives tumorigenesis and progression remain poorly understood. This knowledge gap underscores the need for single‐cell resolution analyses to dissect the interactions between different cell types within the TME. For example, T cell exhaustion can be mediated by tumour cells, tumour‐related macrophages (TAMs) and stromal cells by activating co‐inhibitory receptors such as PD‐1, CTLA‐4 and TIM‐3, and Tregs can secrete immunosuppressive cytokines [16]. Regulatory T cells (Tregs), which secrete immunosuppressive cytokines, further contribute to an immunosuppressive TME, limiting the efficacy of current therapies [17, 18]. Tumour cells themselves exhibit diverse phenotypes, with subsets displaying resistance to chemotherapy and targeted therapies. This extensive heterogeneity poses a significant challenge for precision medicine, as traditional bulk sequencing approaches fail to capture the full spectrum of cellular diversity within tumours [19]. Single‐cell RNA sequencing (scRNA‐seq) has emerged as a powerful tool for dissecting tumour heterogeneity and characterising the TME at unprecedented resolution. By isolating and sequencing individual cells, scRNA‐seq enables the construction of comprehensive cellular atlases, revealing unique gene expression profiles and functional states within tumour populations. This technology has been widely applied to study tumour biology, immune infiltration and developmental processes, providing critical insights into the molecular mechanisms driving cancer progression [20, 21].

Despite these advances, scRNA‐seq has not yet been applied to comprehensively characterise the TME in HB. In this study, we leverage scRNA‐seq to analyse four tissue samples from a single HB patient, including three tumour regions and adjacent normal tissue. Our goal is to provide a detailed landscape of the cellular and molecular heterogeneity within HB tumours, with a focus on identifying key drivers of tumour progression and potential therapeutic targets. We hypothesise that distinct regions of the same tumour exhibit varying degrees of malignancy and immune cell composition, which may underlie the observed differences in treatment response and patient outcomes. Our findings reveal significant intratumoral heterogeneity in HB, with the T1 region showing the highest malignancy and overexpression of HMGB2 and TOP2A. We also identify a diverse immune microenvironment enriched with regulatory T cells (Tregs) and CD8+ effector memory T cells (Tem), suggesting potential mechanisms of immune evasion. Notably, CTLA‐4 and PD‐1 are highly expressed in Tregs and Tem cells, highlighting their potential as immunotherapy targets. This study provides the first comprehensive single‐cell atlas of HB, offering new insights into its biology and paving the way for precision medicine approaches to improve patient outcomes.

## 2 Results

### 2.1 Heterogeneous Nodules and Single‐Cell Expression Atlas Exist in One Hepatoblastoma

**FIGURE 1:**
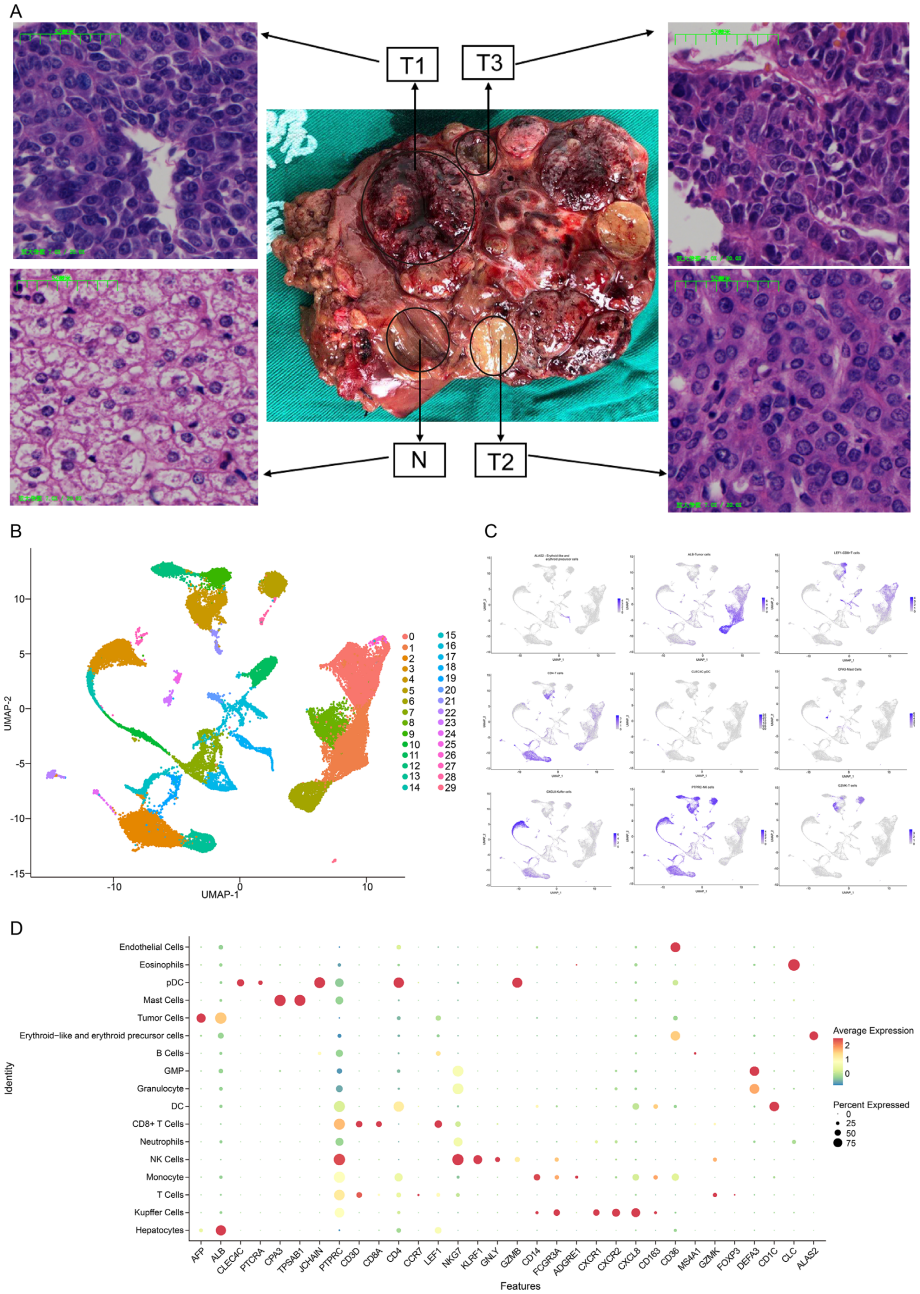
Samples HE staining and single‐cell expression atlas. (A) Showed nuclear atypia, hyperchromatic nuclei and more mitotic cells than others in haematoxylin–eosin (HE) staining. T3 nodule, showed large nucleus, hyperchromatic and mitotic cells, which was less stained than T1. The cell nucleus of T2, was smaller than T1 and T3, and there are relatively few mitotic cells than T1 and T3. (B) UMAP showed the associated clusters for all samples. (C) UMAP plot colour‐coded for expression (blue to grey) of marker genes for the cell types defined above each panel, for hepatocytes, pDC, mast cells, kupffer cells, erythoid‐like and erythroid precursor cells. (D) The dot plot of the expression level of representative well‐known biomarkers across distinct cell types. The *x*‐axis indicated specific biomarkers of each cell subgroup and the *y*‐axis indicated distinct cell subtypes. T1, T2 and T3: Tumour tissue samples; N: Normal tissue adjacent to the carcinoma.

Multiple nodules in different gross morphologies were observed in the tumour section. The adjacent normal liver tissue and three tumour nodules were selected to investigate the heterogeneity of HB (Figure 1). Fresh tissues were immediately processed for single‐cell sequencing (*N*, T1, T2 and T3). Histopathological analysis indicated that T1, T2 and T3 were of embryonal type, fetal type and mixed epithelial‐mesenchymal type, respectively, while normal liver features were observed in the *N* sample (Figure 1A). We acquired single‐cell transcriptomes of 34,063 cells from the three HB tumours and 9529 cells from normal tissue, and identified 30 clusters according to canonical cellular markers from all four samples (Figure 1B). Twenty‐six clusters were identified in normal tissue, while 16, 26 and 23 clusters were identified in T1, T2 and T3, respectively (Figure S1). The representative marker genes for each cluster of cells were shown in Figure S2. Then, we defined the cell types of each cluster according to the informative principal components (*n* = 17). The clusters could be assigned to several cell lineages including parenchymal cells (tumour cells and hepatocytes), lymphoid, myeloid and erythroid precursor cells. In most cases, well‐known markers were used to define the cell clusters, such as CD3D, CD8A and CD4 for T cells, CD19 for B cells and KLRF1 for NK cells [22]. Additional markers, including ALB for hepatocytes, CLEC4C and CD4 for pDC, CPA3 for Mast cells, CXCR2 and CXCL8 for Kupffer cells, and ALAS2 for erythroid‐like and erythroid precursor cells, were also applied (Figure 1C,D). These findings demonstrate significant intratumoral heterogeneity in HB, with T1 showing the highest malignancy and distinct molecular features, suggesting regional differences in tumour progression.

### 2.2 Heterogeneity Analysis of Parenchyma Cells in Normal and Tumour Tissues

**FIGURE 2:**
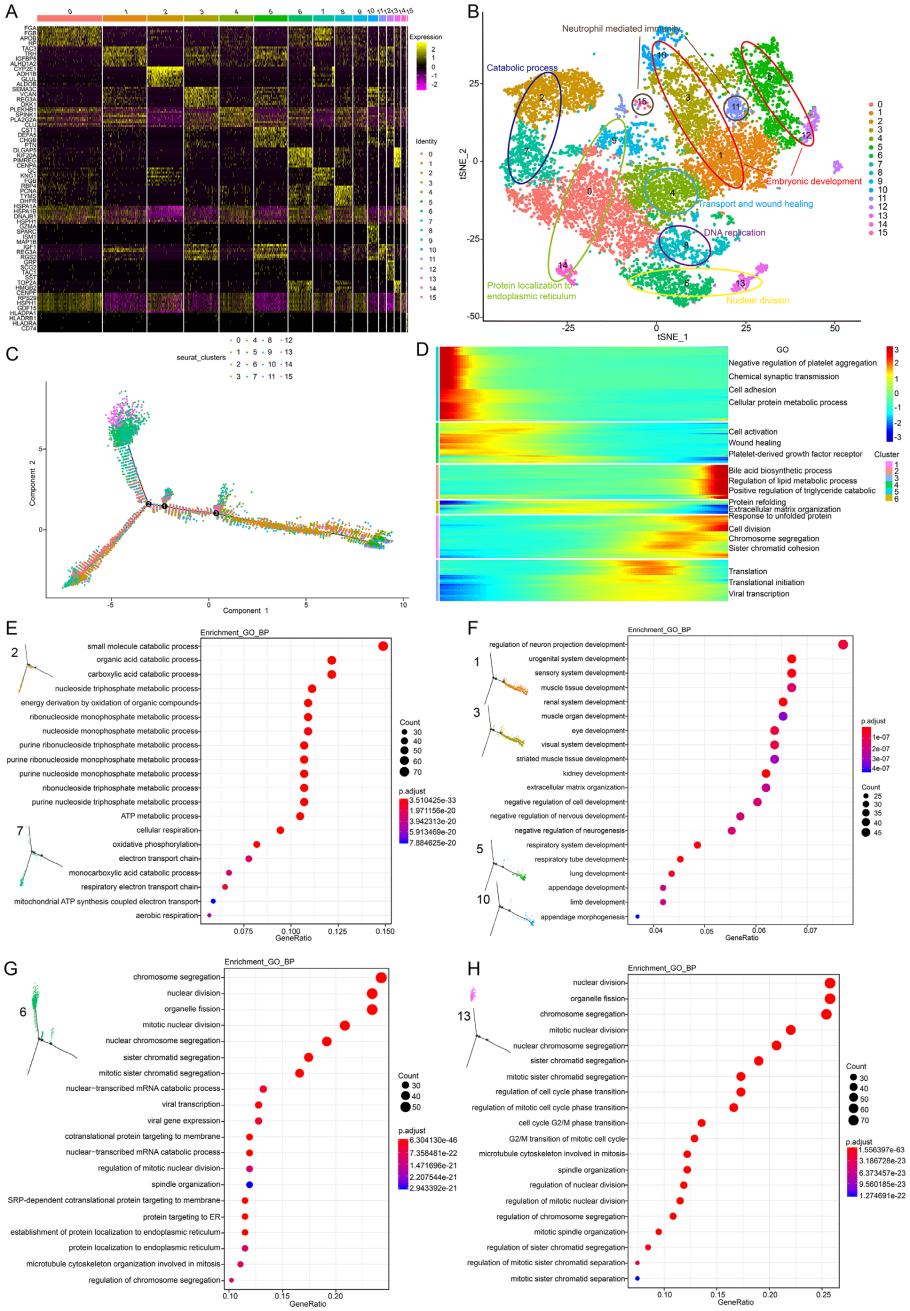
Heterogeneity of parenchyma cells. (A) Heatmap showing parenchyma cells divided into 16 clusters and each cluster's markers. (B) T‐SNE showing different clusters with their related biological types, colour‐coded by their associated cluster; each point represents one cell. (C) Trajectory analysis of the parenchymal cells. (D) According to expression of markers changes, the entire differentiation trajectory can be divided into six clusters, each with different biological functions. (E) Clusters 2 and 7 functions were related to metabolic processes. (F) Clusters 1, 3, 5, 10 and 12 functions were related to embryonic development. (G) Cluster 6 function was related to nuclear division. (H) Cluster 13 functions were related to nuclear division and cell proliferation. GO, Gene Ontology.

A total of 13,180 parenchyma cells including hepatocyte and tumour cells were identified and divided into 16 clusters according to the canonical markers (Figure 2A). According to the expression of markers and biological functions, we marked clusters with similar functions (Figure 2B). Pseudotime analysis was performed on all parenchyma cell clusters to map their differentiation trajectories (Figure 2C). There are specific markers that increase or decrease at different times of the pseudotime differentiation trajectory; according to changes in the expression of markers, the entire differentiation trajectory can be divided into six clusters, each with different biological functions (Figure 2D).

Based on the position of each cluster in the pseudotime differentiation trajectory and the distribution position of each cluster in the tSNE map, we conducted a biological function enrichment analysis for all clusters. We found that parenchyma cells could be classified into distinct functional categories, including metabolism, embryonic development, and cell division (Figure 2B). Clusters 2 and 7 were distributed in similar positions in the pseudotime differentiation trajectory, and their functions were related to metabolism (Figure 2E). Clusters 1, 3, 5, 10 and 12 were mostly concentrated at the beginning of the pseudotime differentiation trajectory, and their functions were related to embryonic development (Figure 2F). Clusters 0, 9 and 14 were tumour cells, and their functions were related to metabolism and protein localisation in the endoplasmic reticulum. Clusters 4 and 8 were tumour cells, but the biological function of cluster 4 is related to transportation and wound healing, while the function of cluster 8 is related to DNA replication. Clusters 6 and 13 were also tumour cells; they were related to cell nuclei division (Figure 2G,H), and they had more malignant and stronger proliferation ability than cluster 8. Cluster 13 was exclusively expressed in T1, and the highly expressed markers TOP2A, CENPF, TPX2 and HMGB2 are related to tumour proliferation and malignancy (Figure 2A) [23]. These findings underscore the dynamic nature of HB progression, with T1 tumour cells representing the most aggressive phenotype.

### 2.3 Intercellular Communication Networks and Gene Regulatory Network in Hepatoblastoma

Using the CellChat tool, we mapped the extensive communication networks among various cell types, including immune cells, tumour cells and stromal components. The most significant interactions were observed between hepatocytes and tumour cells, followed by interactions involving dendritic cells (DCs), monocytes, and Kupffer cells (Figure S3A–C). Key signalling pathways, such as TGF‐β, WNT, and chemokine signalling, were found to be highly active between tumour cells and immune cells. Notably, regulatory T cells (Tregs) exhibited strong interactions with CD8^+^ effector memory T cells (Tem) via the PD‐1/PD‐L1 and CTLA‐4 pathways, suggesting potential mechanisms of immune evasion. These findings highlight the complex communication network within the TME and provide insights into potential therapeutic targets for disrupting tumour–immune cell interactions. In addition, we identified specific ligand–receptor pairs that play critical roles in mediating intercellular communication. For example, neutrophils and NK cells showed significant interactions with tumour cells through pathways such as MIF‐(CD74 + CXCR4) and ANNEXIN‐(FPR1 + FPR2), which are known to modulate immune responses and tumour progression. These results underscore the importance of cell–cell communication in shaping the immune landscape of HB and suggest that targeting these interactions could enhance anti‐tumour immunity.

To further elucidate the transcriptional regulatory networks driving HB progression, we utilised the SCENIC pipeline for single‐cell regulatory network inference. This analysis identified key transcription factors (TFs) and their target genes (regulons) that are active in various cell types within the tumour microenvironment (Figure S4). For example, tumour cells exhibited high activity of regulons regulated by TFs such as GATA2, NFIL3 and E2F1, which are associated with cell proliferation and survival. In contrast, immune cells, particularly CD8+ T cells and NK cells, displayed active regulons linked to immune responses and cytokine signalling pathways, including those regulated by IRF9 and BATF3. Endothelial cells were found to express significant levels of SMAD9 and TFE3, indicating their involvement in signalling pathways related to angiogenesis. Additionally, we observed that B cells and dendritic cells (DCs) showed distinct TF activities, with BCL6B and ZNF212 being prominent in B cells, while GFI1 was notably active in DCs. The identification of these key TFs and their interactions underscores the complex regulatory landscape of HB and highlights potential therapeutic targets. Furthermore, the observed interactions between immune and tumour cells, including those involving transcription factors such as MYB and SOX6, emphasise the interplay between transcriptional regulation and cell communication in shaping the immune landscape of HB.

### 2.4 The Pathological Type and HMGB2 Level Were Associated With HB Prognosis

**FIGURE 3:**
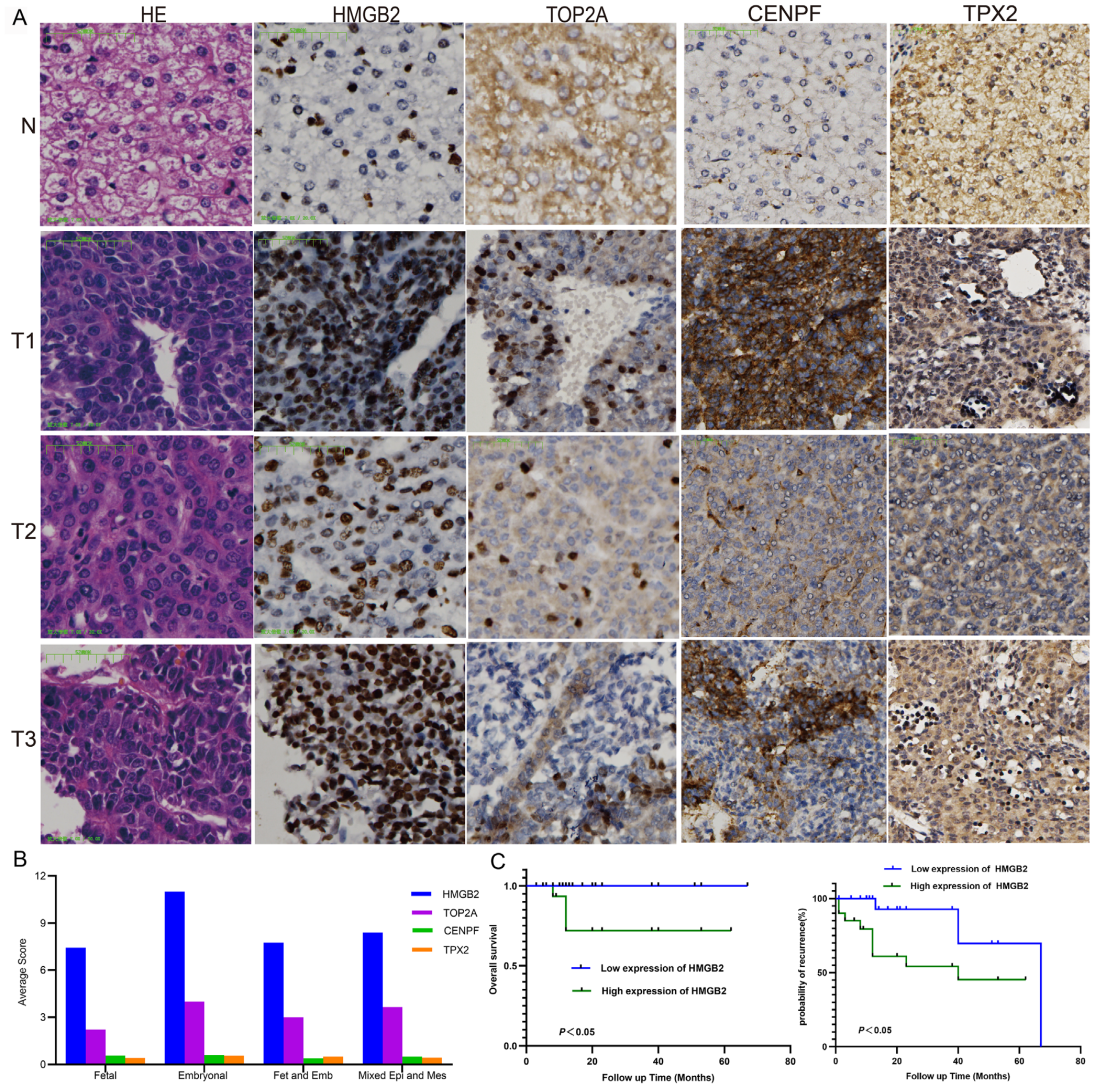
HE staining and tumour cell markers immunohistochemistry in four samples. (A) Four sample HE staining, HMGB2, TOP2A, CENPF and TPX2 immunohistochemistry. (B) In 40 samples, tumour cell marker expression levels in four HB pathological types. (Fetal: Fetal type; Embryonal: Embryonal type, Fet and Emb: Fetal and Embryonal; Mixed Epi and Mes: Mixed epithelial‐mesenchymal type). (C) The Kaplan–Meier survival analysis of HB patients demonstrated that high HMGB2 levels correlate with significantly worse patient survival rates and high recurrence. (Pictures were magnified 200×).

We employed HE staining and immunohistochemistry (IHC) to assess the degree of differentiation and malignancy in the four samples. HE staining revealed that tumour cells in T1 exhibited nuclear atypia, hyperchromatic nuclei, and a higher number of pathologic mitotic figures compared to other regions (Figure 3A). IHC analysis demonstrated that tumour cell markers, including HMGB2, TOP2A, CENPF, and TPX2, were strongly expressed in T1, with HMGB2 showing higher expression than TOP2A (Figure 3A). In 40 HB samples, HMGB2 expression was significantly higher in embryonal‐type tumours compared to other pathological types (Figure 3B). The Kaplan–Meier survival analysis of HB patients showed that high HMGB2 expression levels were associated with worse survival rates in HB, and the high expression of HMGB2 was associated with high recurrence (Figure 3C). TOP2A was mostly positive or moderately positive in tumours; it was not correlated with HB overall survival rates. Forty samples of HB patients IHC showed that in embryonal type CENPF and TPX2 were expressed more than in other pathological types; the high expressions of CENPF and TPX2 were not associated with HB overall survival rates. These results suggest that HMGB2 may serve as a potential prognostic marker for HB, with high expression correlating with poor clinical outcomes, providing a basis for future therapeutic targeting. This underscores the importance of HMGB2 as a key driver of HB progression and highlights its potential as a therapeutic target.

### 2.5 Tumour Microenvironment and Novel Immunotherapy Targets for HB

**FIGURE 4:**
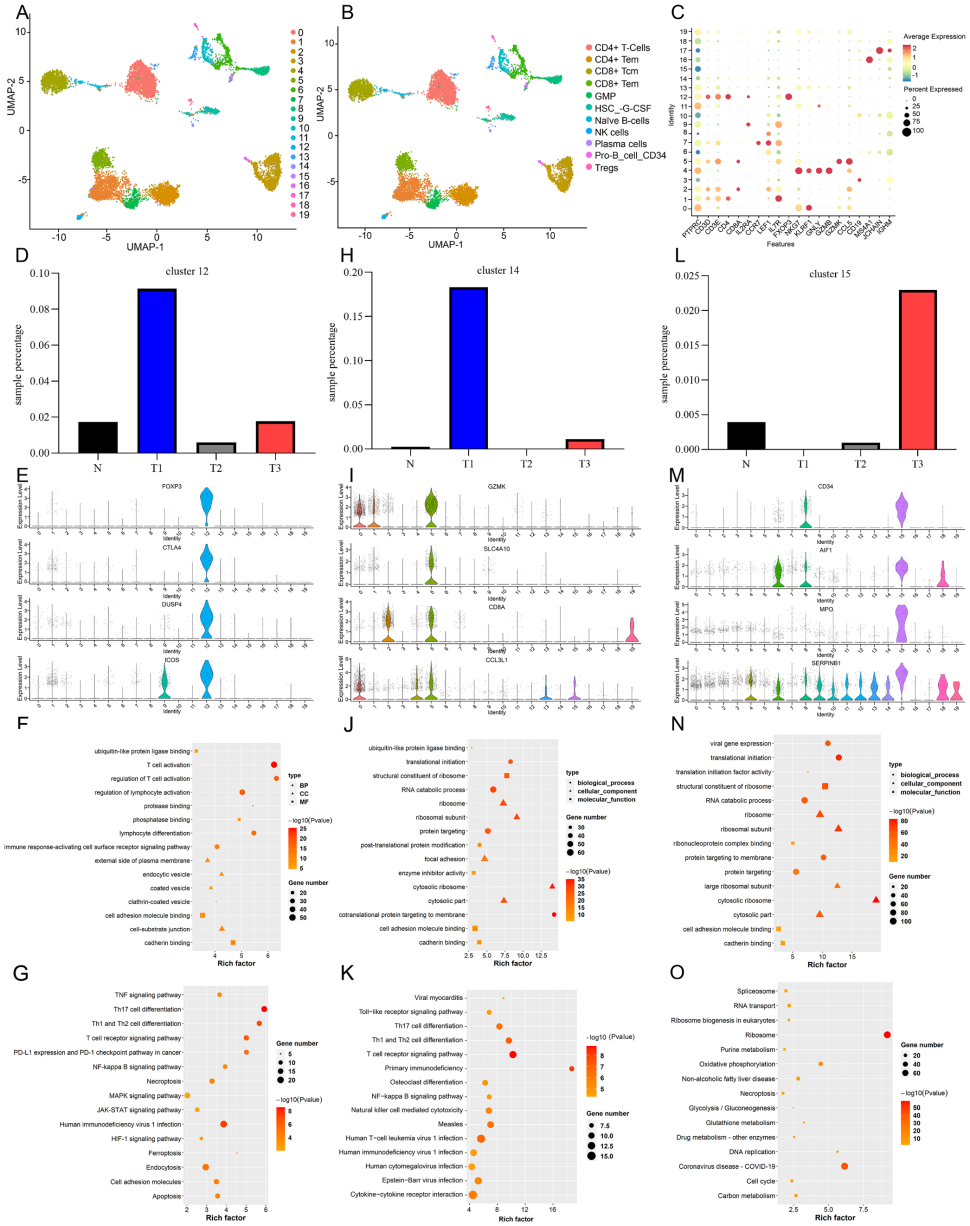
Lymphocyte clusters in adjacent tumour normal tissue and tumour tissues. (A, B) The UMAP plot of lymphocyte cell clusters and cell types. (C) Dot plot showing the most characteristic marker genes for each cluster. (D) The cluster 12 (Tregs) percentage in four samples. (E) Violin plots showing the expression distribution of selected genes for Tregs. (F, G) Differences in enrichment pathway activities of Treg cells. (H) Percentage of cluster 14 (CD8 + Tem) in four samples. (I) Violin plots showing expression marker genes for CD8 + Tem through logFC. (J, K) Differences in function enrichments of CD8 + Tem. (L) The cluster 15 (GMP) percentage in four samples. (M) Violin plots showing expression marker genes for GMP of selected genes through logFC. (N, O) function enrichments about cluster 15.

A total of 10,532 lymphoid cells were detected and re‐clustered into 20 clusters, which were further annotated into 11 cell types (Figure 4A,B). These included natural killer cells (NKG7 and KLRF1, clusters 0, 4, and 11), CD8+ central memory T cells (Tcm, CD8A, clusters 2 and 19), CD8+ effector memory T cells (Tem, GZMK, clusters 5, 13, and 14), CD4+ Tem (CD4 and IL7R, clusters 1 and 9), CD4+ regulatory T cells (Tregs, CTLA4 and FOXP3, cluster 12) and naïve B cells (CD19 and MS4A1, clusters 3 and 16) (Figure 4C). The cluster 12 (FOXP3, CTLA4) was CD4 + Tregs; it in T1 sample had the highest expression (Figure 4D,E). The cluster 12 function enrichments included regulation of T cell activation and regulation of lymphocyte activation; its activated signalling pathways were PD‐L1 expression and PD‐1 checkpoint pathway in cancer, Human immunodeficiency virus 1 infection and T cell receptor signalling pathway (Figure 4F,G). The cluster 14 was CD8 + Tem; it was mainly expressed in T1 samples, a highly immunity effector and proliferative cluster (Figure 4H), the markers were GZMK, SLC4A10, CD8A (Figure 4I). The function enrichments of CD8 + Tem mainly included T cell receptor signalling pathway, Th1 and Th2 cell differentiation and natural killer cell mediated cytotoxicity (Figure 4J,K). The cluster 15 was GMP; its markers included CD34, AIF1, MPO, SERPINB1; it was mainly expressed in T3 sample (Figure 4L,M), function enrichments mainly included immune response required for pathogen clearance such as viral gene expression, protein targeting to membrane and Coronavirus‐COVID‐19 (Figure 4N,O). These findings highlight the complex immune microenvironment in HB, with Tregs and CD8+ Tem cells playing central roles in immune evasion and tumour progression, suggesting that immune checkpoint blockade may be a promising therapeutic strategy.

**FIGURE 5:**
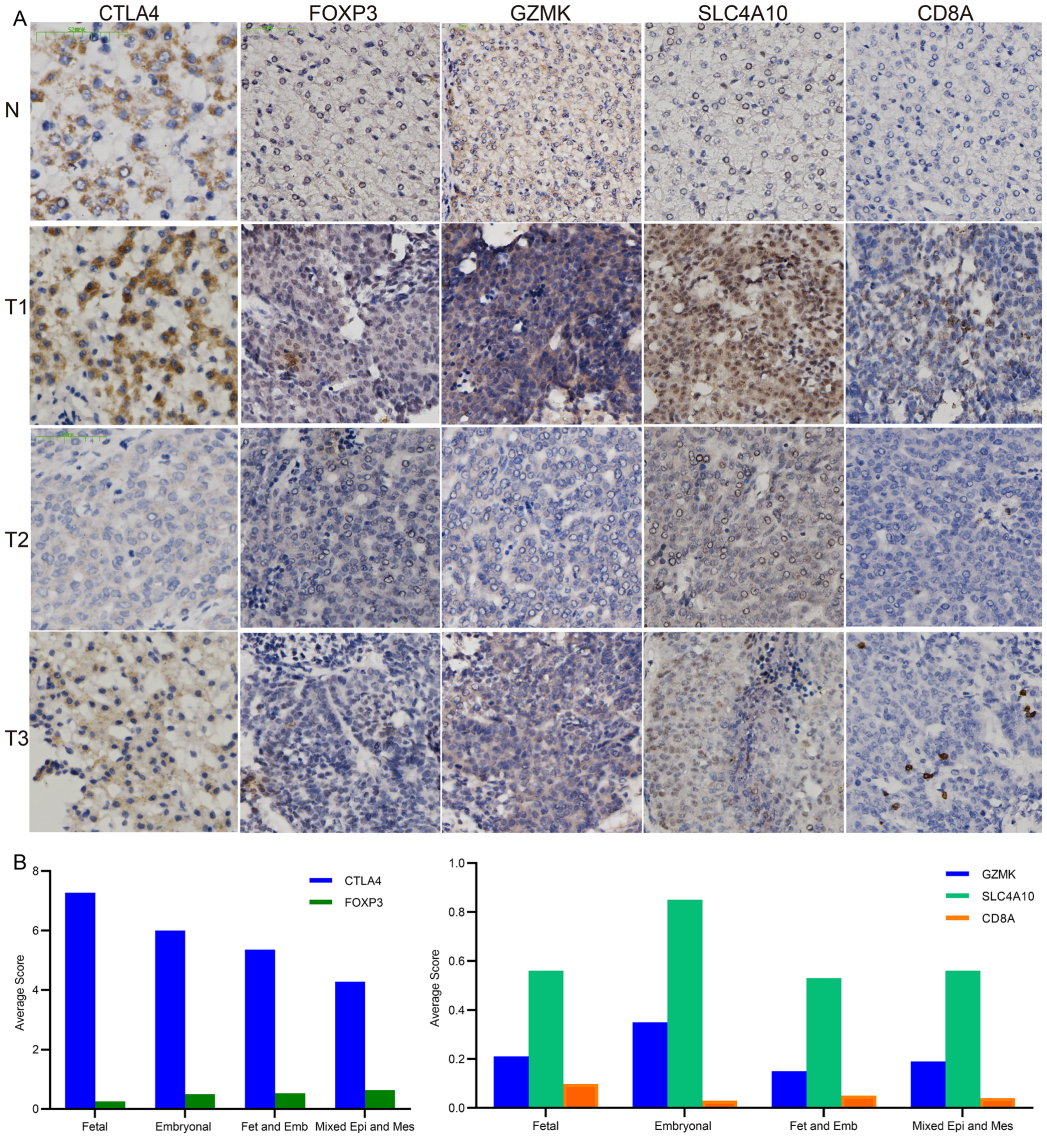
Immunohistochemistry of lymphocyte cell markers and expression levels in different pathological types (A) Lymphocyte cell marker immunohistochemistry in four samples. (B) The CD4 + Treg cell markers CTLA4, FOXP3 and CD8 + Tem markers GZMK, SLC4A10 and CD8A expression levels in four pathological types. (Fetal: Fetal type; Embryonal: Embryonal type, Fet and Emb: Fetal and Embryonal; Mixed Epi and Mes: Mixed epithelial‐mesenchymal type). (Pictures were magnified 200×).

CTLA4 exhibited cytoplasmic immunostaining in both adjacent normal tissues and tumor cells, with particularly strong expression observed in T1‐stage tumor specimens. (Figure 5). From 40 samples collected, we can acknowledge that CTLA4 in all pathological types was expressed, and the highest expression was in fetal (Figure 5B). The CD4 + Treg cell marker FOXP3 was positive staining in T1, weak staining in T2 and T3, but the expression level was higher in embryonal and mixed epithelial‐mesenchymal types than in other types (Figure 5B). For CD8+ Tem cells, markers such as GZMK, SLC4A10 and CD8A were strongly positive in T1, with weaker staining in T2 and T3. While CD8A expression was higher in fetal‐type tumours, GZMK and SLC4A10 were more highly expressed in embryonal‐type tumours (Figure 5B). The enrichment of Tregs and CD8+ Tem cells in the TME indicates an immunosuppressive environment, with CTLA‐4 and PD‐1 representing promising targets for immunotherapy. These findings highlight the potential of immune checkpoint blockade as a therapeutic strategy for HB, particularly in tumours with high Treg and CD8+ Tem cell infiltration.

### 2.6 The Myeloid Cells for HB

**FIGURE 6:**
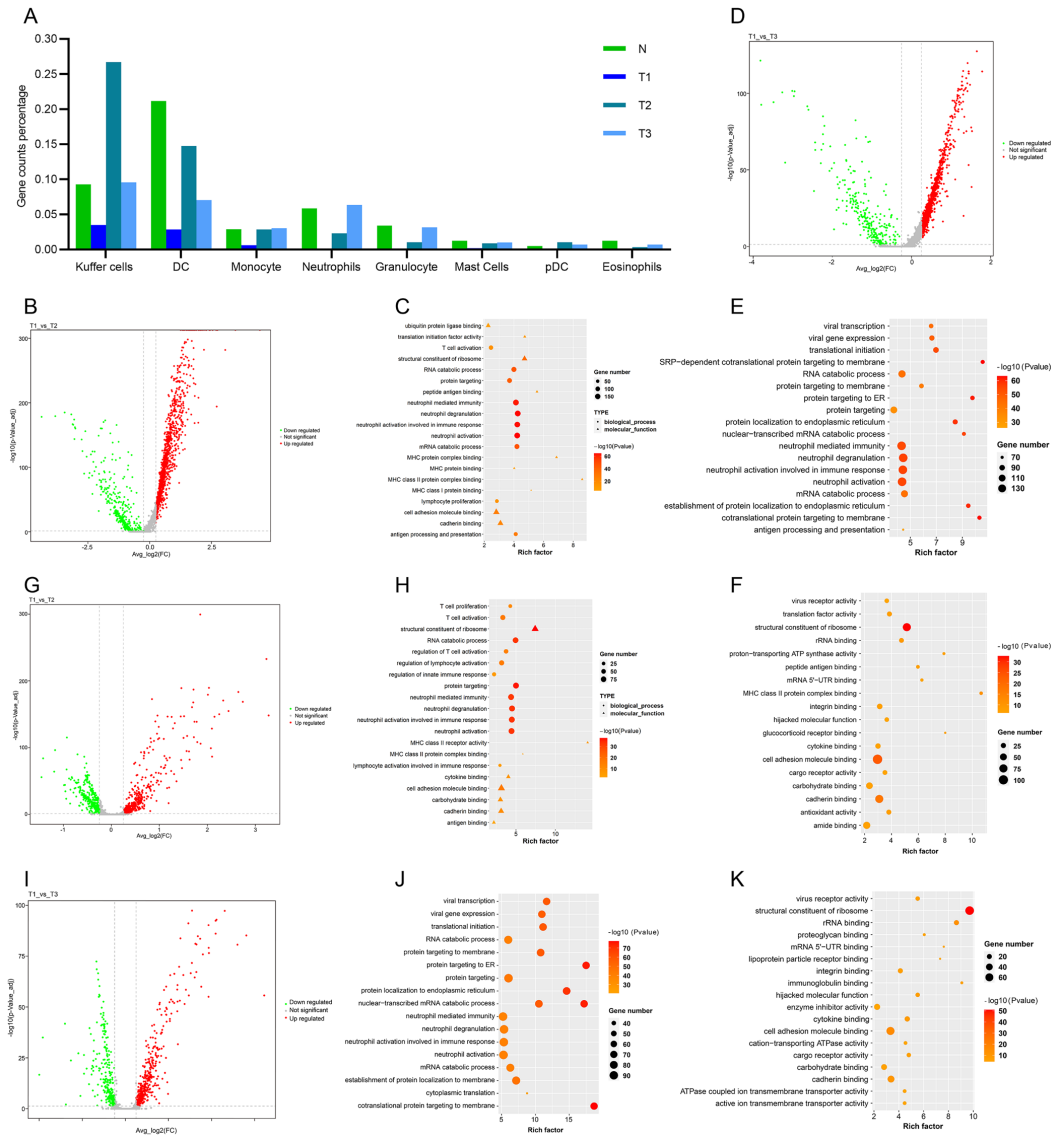
Distinct myeloid cells in HB tumour tissues. (A) The proportion of myeloid cells in each sample. (B) DEGs between T1 and T2 in Kuffer cells. (C) The GO analysis of T1 versus T2 in Kuffer cells. (D) DEGs between T1 and T3 in Kuffer cells. (E, F) The GO analysis of T1 versus T3 in Kuffer cells. (G) DEGs between T1 and T2 in DC. (H) The GO analysis of T1 versus T2 in DC. (I) DEGs between T1 and T3 in DC. (J, K) The GO analysis of T1 versus T3 in DC. GO: Gene Ontology; BP: Biological process; MF: Molecular Function; DEGs: Differentially expressed genes.

Myeloid cells play critical roles in the antigen‐presentation and inflammation responses. These cell types were typically less abundant in T1 than T2 or T3 tissues; Kuffer cells and DC were detected in similar numbers. The largest difference in Kuffer cells was between T1 and T2 samples (Figure 6A). T versus *N*, T1 versus T3 and T2 versus T3 also have many DEGs (Figure 6B,D and Figure S5A,D). These DEGs showed similar biological processes and molecular functions including T cell activation, neutrophil mediated immunity and MHC class protein binding (Figure 6C,E,F and Figure S5B,C,E,F). The largest difference in DC was also between T1 and T2 samples (Figure 6G). Multiple genes were found to be differentially expressed in T1 versus T2 and T1 versus T3 (Figure 6G,I). However, the DEGs of T versus *N* and T2 versus T3 were less than that (Figure S5G,J). All functions mainly enriched in T cell activation, neutrophil mediated immunity and neutrophil activation (Figure 6H,J,K and Figure S5H,I,K,L). These results indicate that myeloid cells play a critical role in shaping the immune landscape of HB, with T3 exhibiting a more active immune microenvironment compared to other regions. This suggests that myeloid cells may contribute to the immune response against HB and could be targeted to enhance anti‐tumour immunity.

**FIGURE 7:**
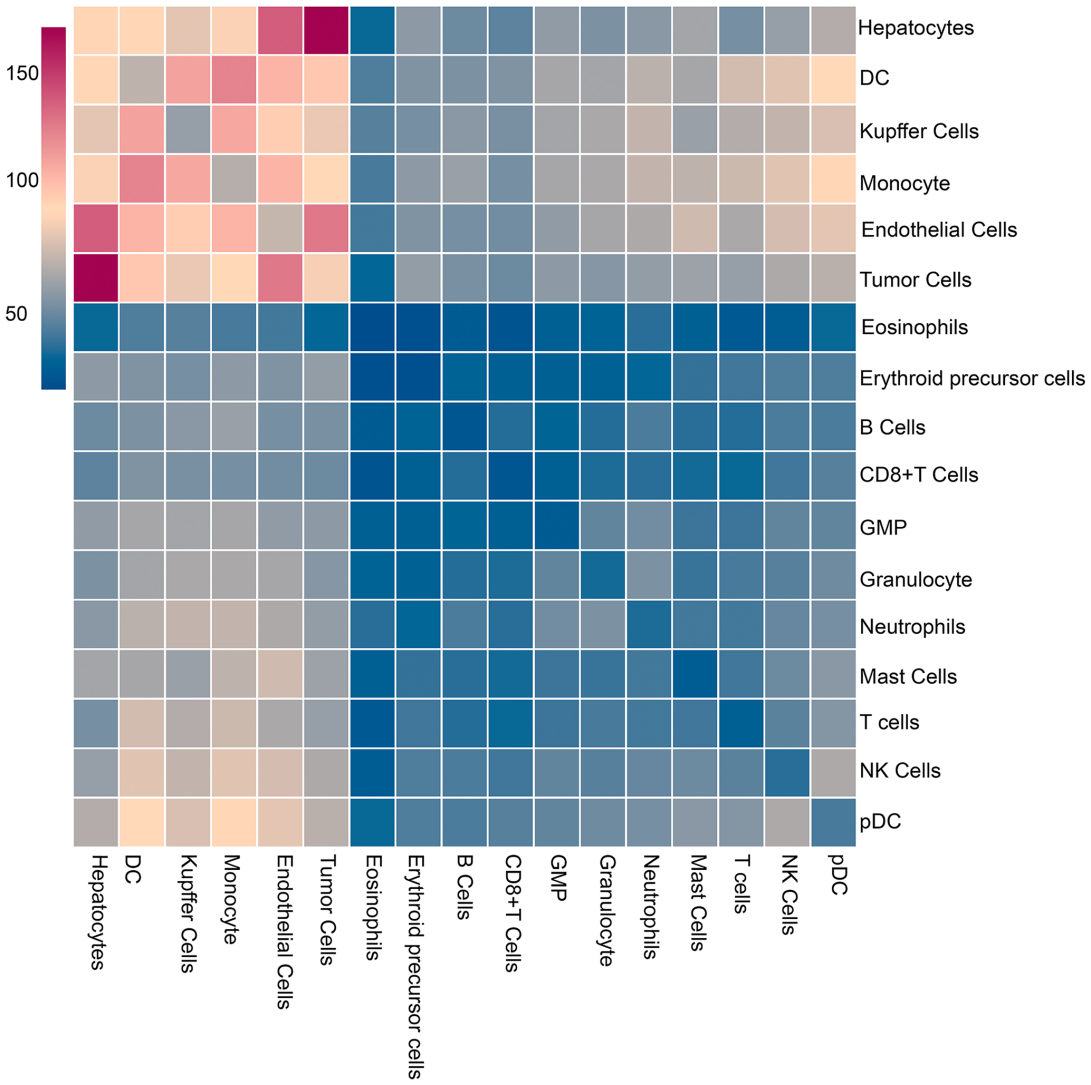
Intercellular ligand–receptor interactions in HB.

The ligand‐receptor interaction between hepatocytes and tumour cells is the most significant, followed by the interactions between hepatocytes and tumour cells with DC, monocytes and Kuffer cells (Figure 7). Intercellular communication within the TME plays a crucial role in HB progression, highlighting potential therapeutic targets for disrupting tumour‐immune cell interactions.

## 3 Discussion

Hepatoblastoma (HB) is a malignant liver tumour that predominantly affects children under 5 years of age. The pathogenesis of HB is complex, involving multiple genetic and environmental factors. Our study leverages single‐cell RNA sequencing (scRNA‐seq) to dissect the intricate cellular landscape of HB, revealing significant insights into the genetic and phenotypic diversity within individual tumours. The results highlight a substantial degree of intratumoral heterogeneity, as each tumour region (T1, T2 and T3) displayed distinct gene expression profiles and cellular compositions, consistent with previous findings that underscore the role of intratumoral heterogeneity in treatment resistance across various cancers [24, 25].

One of the key challenges in HB research is understanding how this heterogeneity impacts therapeutic efficacy. Our findings indicate that different degrees of differentiation exist within different areas of a single tumour, a phenomenon that has been reported in other paediatric malignancies such as neuroblastoma and Wilms tumour, where heterogeneous cell populations are associated with poor prognosis and treatment resistance [26, 27]. This is critical because it suggests that current treatment modalities may selectively kill only a fraction of tumour cells, allowing drug‐resistant populations to survive and subsequently drive disease recurrence. The heterogeneity we observed supports the notion that effective treatment strategies must account for the diverse cellular makeup of tumours, as therapeutic agents may target only a subset of tumour cells, potentially leading to the emergence of resistant clones.

Moreover, scRNA‐seq technology has rapidly advanced our understanding of tumour biology, facilitating the identification of resistance mechanisms and patterns of immune infiltration [28]. In our analysis, we found that the activation pathways related to COVID‐19 were present in transitional states of certain cells, highlighting a potential connection between viral infection and HB tumour progression [29]. The expression of ACE2 in hepatocytes is particularly noteworthy, suggesting that the liver may be a potential target for SARS‐CoV‐2, raising important considerations for paediatric patients with HB amid the ongoing pandemic [30, 31]. This connection between viral infections and cancer progression underscores the need for further investigations into how such infections may influence tumour biology and treatment outcomes.

In our study, T1 exhibited the worst degree of differentiation, with the predominant cell type being tumour cells expressing signature markers such as TOP2A and HMGB2. The enrichment of pathways like Wnt signalling and cell cycle regulation indicates a complex interplay of molecular mechanisms driving HB progression [32, 33, 34, 35]. Similar observations have been reported in other studies where the upregulation of Wnt signalling has been implicated in promoting tumorigenesis and resistance to chemotherapy in various cancers, including colorectal cancer [36, 37, 38]. TOP2A has been established as a therapeutic target in various malignancies, and its increased expression in T1 compared to T2 and T3 may suggest its role in promoting tumour cell proliferation through the inhibition of p53 pathways [39, 40, 41, 42]. In line with our findings, other studies have also reported that elevated TOP2A expression correlates with poor prognosis in several cancers, including hepatocellular carcinoma [43].

Additionally, HMGB2, which was notably overexpressed in T1, has been linked to poor prognostic outcomes in various malignancies, facilitating tumour cell proliferation and invasion through the p38 MAPK signalling pathway [44]. The identification of these markers as potential therapeutic targets for HB is a promising avenue for future research, echoing findings from other studies that have proposed similar therapeutic strategies based on the modulation of these pathways in different tumour types [45].

The implications of our findings suggest that combining targeted therapies with immunotherapy may provide a promising approach for treating HB. Recent advancements in cancer immunotherapy have shown great potential in improving the prognosis of various malignancies [46]. Immune checkpoint inhibitors, such as those targeting CTLA‐4 and PD‐1, have demonstrated significant clinical responses in other cancers, and our study identifies high expression of Treg markers like FOXP3 and CTLA‐4 in HB, suggesting these may serve as important immunotherapeutic targets [47, 48, 49, 50]. Furthermore, the presence of CD8+ memory T cells, particularly GZMK+ CD8 + Tem cells, in the tumour microenvironment underscores the potential for harnessing the immune system to combat HB [51, 52, 53, 54, 55, 56]. These observations resonate with findings from studies in adult cancers, where T cell infiltration is associated with a better prognosis and response to immunotherapy [57].

Despite the significant insights provided by our study, there are several limitations. First, the sample size is relatively small, and we did not apply single‐cell data correction methods. This limitation may affect the generalisability of our findings and the statistical power to detect significant associations [58]. Future research should aim to increase the sample size to validate these results and further investigate the functional roles of TOP2A and HMGB2 in the pathogenesis and treatment resistance of hepatocellular carcinoma (HB). Additionally, the mechanisms underlying the observed heterogeneity in tumour cells need to be elucidated, which could inform the development of combination therapies targeting multiple pathways simultaneously. Furthermore, in tumour data analysis, more advanced deep learning algorithms should be employed to achieve better predictions, allowing for a more thorough analysis of important coding and non‐coding genes in tumours [59, 60, 61]. Finally, transcription factor‐related transcriptional regulation plays a crucial role in tumour progression, and incorporating this aspect into future studies will be essential for a comprehensive understanding of the molecular mechanisms of cancer [62, 63, 64, 65].

In conclusion, our work provides a valuable resource for understanding the complex gene expression landscape of heterogeneous cell types in HB. We identified potential mechanisms underlying intratumoral heterogeneity and tumour progression while offering a preliminary understanding of the immune microenvironment of HB. Addressing the limitations of our study and validating these findings in larger cohorts will be crucial for advancing the precision medicine approach in HB, ultimately improving clinical outcomes for affected patients. Continued investigation into the interplay between tumour heterogeneity, immune response, and therapeutic resistance will be essential in the quest for more effective treatment strategies for hepatoblastoma.

## 4 Materials and Methods

### 4.1 Human Tissue Specimen

A 1‐year‐old female child with HB underwent surgical resection at the Affiliated Hospital of Qingdao University. Four samples from one tumour tissue (including three tumour samples and one adjacent non‐tumour sample) were obtained under the supervision of a qualified pathologist. This study was approved by the Ethics Committee of the Affiliated Hospital of Qingdao University, and the informed consent of patients' guardians was obtained.

### 4.2 Single Cell Suspension and Quality Control (QC)

To explore the cellular diversity in HB, following resection, one non‐malignant liver tissue sample from a distal region within the same liver lobe and three tumour tissue samples were obtained, rapidly digested to a single cell suspension and analysed using scRNA‐seq involving a micro‐well protocol with unique transcript counting through barcoding with unique molecular identifiers (UMIs). Following gene expression normalisation for read depth and mitochondrial read count, we applied principal component analysis (PCA) (the top 50 principal components with the largest variance were selected) on variably expressed genes across all cells to explore the cellular composition of tumours and identified 30 clusters from four samples.

### 4.3 Single Cell Transcriptome Capturation

The cells were first stained with the fluorescent dye Calcein AM (Thermo Fisher Scientific Cat. No. C1430) and Draq7 (Cat. No. 564904), using the BD Rhapsody scanner to precisely determine cell concentration and viability. Single‐cell transcriptome was captured using the BD Rhapsody Expression system based on Fan et al. [66]. Cells were loaded into a BD Rhapsody cartridge and primed and treated in strict accordance with the manufacturer's protocol (BD Biosciences, Doc ID:210966). The cell capture beads (BD Biosciences) were then overloaded onto the cartridge to ensure that almost every well contained one bead and excess beads were washed out of the cartridge. After the lysis of the cells (BD Biosciences) with lysis buffer, cell capture beads were extracted and washed for downstream experiments.

### 4.4 Library Constructing and Sequencing

After utilising the BD Rhapsody System to obtain the transcriptome of single cells, we generated a cDNA library containing cell markers and unique molecular identifier (UMI) information. Briefly, we generated single‐strand cDNA from the single cell transcriptome captured by microbeads through reverse transcription and exonuclease digestion with the BD Rhapsody cDNA Kit (BD Biosciences, Cat. No. 633773). Through random priming and extension (RPE) PCR and WTA index PCR with the BD Rhapsody WTA Amplification Kit (BD Biosciences, Cat No. 633801), we produced the final cDNA library from single strand full‐length cDNA. All the libraries were sequenced in a PE150 mode (Pair‐End for 150 bp read) on the NovaSeq platform (Illumina). After cell type annotation, we performed single cell pseudotime analysis for each cell type separately using Monocle2 (http://cole‐trapnell‐lab.github.io/monocle‐release/) with the DDR‐Tree reduction method. As for non‐immune cells, single cell pseudotime analysis was performed with default parameters to eliminate batch effect. Briefly, the raw gene expression matrix of non‐immune cells was converted into a Monocle object. During feature selection, we chose marker genes of each cluster as ordering genes for downstream trajectory analysis.

### 4.5 Immunohistochemistry

In addition to the sample of this child, we collected 40 cases of paraffin block specimens of HB from the Department of Paediatric Surgery, Affiliated Hospital of Qingdao University from 2014 to 2020. Tissue fixation, dehydration, transparency, wax coating, and tissue embedding: The embedded tissue was taken out of the mould, placed on a paraffin sectioning machine; the slicer was adjusted, wherein the thickness of the section was generally 3–4 μm, using a writing brush to pull the cut paraffin tissue slice outward and tweezers to flatten the slice containing the complete tissue into 40°C warm water. Slides were placed in 40°C water, and the tissue slices were attached to the slides obliquely to avoid the generation of air bubbles. The tissues were heated to expand so that the tissues could not wrinkle; the fished‐out slide was placed on a 70°C baking rack for 10 min. The slices were dried before dyeing: The slides were placed on slide racks in a 60°C oven overnight. Dewaxing: The rack loaded with slides was put into dewaxing solutions I, II and III for 5 min each, and then 100% ethanol, 95% ethanol, 80% ethanol and 70% ethanol were sequentially put in for 2 min each to elute the dewaxing solution from the slides. Washing the slices: The slides were washed successively three times, 2 min each time, in tap water, distilled water and PBS. Antigen retrieval:Perform antigen retrieval with pH 6.0 citric acid buffer by boiling the solution in a pressure cooker.Insert slides, seal the lid, reduce to medium‐low heat after steam release, maintain boiling for 5 minutes, then immediately cool under running water. Water washes: the sections were washed with distilled water three times, 1 min each time. Removal of endogenous peroxidase: endogenous peroxidases were eliminated by heating in a microwave with 3% hydrogen peroxide for 2 min. The slides were washed with distilled water for three times and with PBS 1 time for 1 min. Primary antibodies: Before the addition of the primary antibody, the tissues requiring dropwise addition of the primary antibody were surrounded with an oil pen. The primary antibody was incubated at the dilution concentrations of HMGB2 1:300, TOP2A 1:250 and CTLA4 1:100, respectively, for 1.5 h at room temperature. The slides were washed with PBS three times, 2 min each time. Secondary antibodies: The secondary antibody containing horseradish peroxidase was added dropwise and incubated at room temperature for 20 min. The slides were washed with PBS three times, 2 min each time. DAB chromogen was prepared as follows: 50 μL concentrated solution added to 1 L high‐sensitivity substrate to formulate working solution; tissue sections incubated with DAB for 1‐2 min; color development terminated by tap water rinse; subsequent 5 min flushing under running water. Flush with running water for 5 min. Haematoxylin counterstaining nuclei: First, the slices were put into a stabilising solution for 1 min, taken out and directly put into a haematoxylin dye solution for dip dyeing for 5 min, washed with flowing water, put into an acidic alcohol differentiation solution for differentiation for a few seconds, washed with flowing water, put into an alkaline anti‐blue solution for 2 min to make the nuclei blue, and finally washed with flowing water for 10 min. Tissue dehydration was performed through graded ethanol series (80%, 95%, 100%), 2 min per concentration, followed by xylene clearing. Sealing the slides: The section sealing rubber was dropped beside the tissue and covered with a cover glass; care was taken to avoid the generation of bubbles. Drying at room temperature. All sections were observed and photographed under a scanning electron microscope (200× magnification).

### 4.6 Preprocessing and Quality Control

Raw sequencing reads were processed using the BD Rhapsody Whole Transcriptome Assay Analysis Pipeline. Low‐quality reads were filtered out, and the remaining reads were mapped to the human reference genome (GRCh38) using STAR (version 2.5.2b). Unique molecular identifiers (UMIs) were used to correct for amplification bias, and a single‐cell expression matrix was generated. Cells with fewer than 200 detected genes or more than 10% mitochondrial reads were excluded from further analysis.

### 4.7 Data Normalisation and Dimensionality Reduction

The gene expression matrix was normalised using the Seurat R package (version 3.6.0) [67]. Briefly, gene expression counts were normalised to the total UMI count per cell, and log‐normalisation was applied. The top 2000 highly variable genes were selected for downstream analysis. Principal Component Analysis (PCA) was performed to reduce dimensionality, and the top 25 principal components were selected based on the elbow plot and Jackstraw analysis. *t*‐distributed stochastic neighbour embedding (*t*‐SNE) was used for further dimensionality reduction and visualisation of cell clusters.

### 4.8 Cell Type Annotation

Cell clusters were annotated based on the expression of canonical marker genes. For example, T cells were identified by the expression of CD3D, CD4 and CD8A; B cells by CD19 and MS4A1; and myeloid cells by CD14, CD68 and CD163. Hepatocytes were identified by the expression of ALB and APOA1. Cell type annotation was further validated using known marker genes from the literature.

### 4.9 Pseudotime Analysis

Pseudotime analysis was performed using Monocle2 (http://cole‐trapnell‐lab.github.io/monocle‐release/) to map the differentiation trajectories of parenchymal cells. The top 2000 highly variable genes were used for trajectory inference, and the DDR‐Tree reduction method was applied to construct the differentiation trajectory. Cells were ordered along the pseudotime axis based on their gene expression profiles, and clusters were defined based on the expression of key marker genes.

### 4.10 Cell–Cell Communication Networks in HB

Cell–cell communication networks were analysed using the CellChat R package. Ligand‐receptor interactions were inferred based on the expression of known ligand‐receptor pairs in the CellChat database. The strength and significance of interactions were calculated, and key signalling pathways (e.g., TGF‐β, WNT, and chemokine signalling) were identified. The results were visualised as network diagrams and heatmaps.

### 4.11 Regulatory Network Analysis Using SCENIC

Single‐cell regulatory network inference was performed using the SCENIC (Single‐Cell Regulatory Network Inference and Clustering) pipeline. Co‐expression modules were identified, and transcription factor (TF) motifs were enriched using the RcisTarget database. Regulon activity was scored using the area under the curve (AUC) method, and the results were visualised as heatmaps and *t*‐SNE plots.

## Author Contributions

**Mingdi Ma:** data curation (equal), writing – original draft (equal). **Chen Jin:** resources (equal), validation (equal), visualization (equal). **Qian Dong:** conceptualization (equal), formal analysis (equal), funding acquisition (equal).

## Conflicts of Interest

The authors declare no conflicts of interest.

## Supporting information


**Figure S1.** Landscape of four samples. (A) The UMAP showing 9529 cells of *N* (non‐tumorous) sample including 26 clusters. (B) The UMAP showing 12,078 cells of T1 (tumour) sample including 16 clusters. (C) The UMAP showing 12,107 cells of T2 (tumour) sample including 26 clusters. (D) The UMAP showing 9878 cells of T3 (tumour) sample including 16 clusters. Colour‐coded by their cluster and every dot present a single cell.

**Figure S2.** Dot plot showing the most characteristic markers for parenchyma cells each cluster.

**Figure S3.** Cell–Cell communication networks in hepatoblastoma. (A) Interaction number among various immune cell types and tumour cells are depicted in the network diagram. (B) Interaction weights and strengths among various immune cell types and tumour cells are depicted in the network diagram. (C) Heatmap depicts the strength of interactions between cell types, with darker colours indicating stronger communication. (D) Statistical significance of the interactions is indicated by *p*‐values, with thresholds set for *p* < 0.01 and 0.01 < *p* < 0.05, providing insights into critical communication pathways within the tumour microenvironment.

**Figure S4.** Transcription factor activity in cells of hepatoblastoma. (A) The heatmap displays the activity of key transcription factors (TFs) and their target genes (regulons) in different cell types within the HB tumour microenvironment. (B) Regulon specificity score (RSS): The bar plot shows the regulon specificity scores (RSS) for each cell type, highlighting the most specific regulons for each cell population.

**Figure S5.** Distinct of myeloid cells in HB tumour tissues. (A) DEGs between T and *N* in Kupffer cells. (B), (C) The GO analysis of T versus *N* in Kupffer cells. (D) DEGs between T2 and T3 in Kupffer cells. (E), (F) The GO analysis of T2 versus T3 in Kupffer cells. G, DEGs between T and *N* in DC. (H), (I) The GO analysis of T versus *N* in DC. J, DEGs between T2 and T3 in DC. (K), (L) The GO analysis of T2 versus T3 in DC. GO: Gene Ontology; BP: biological process; MF: Molecular Function; DEGs: Differentially expressed genes.

## Data Availability

The data that support the findings of this study are available from the corresponding author upon reasonable request.
