# Supplementary figures and images for: Intratumoral Heterogeneity and Immune Microenvironment in Hepatoblastoma Revealed by Single‐Cell RNA Sequencing
Source: J Cell Mol Med. 2025 Mar 18;29(6):e70482. doi: 10.1111/jcmm.70482 (PMC11915626; doi:10.1111/jcmm.70482)

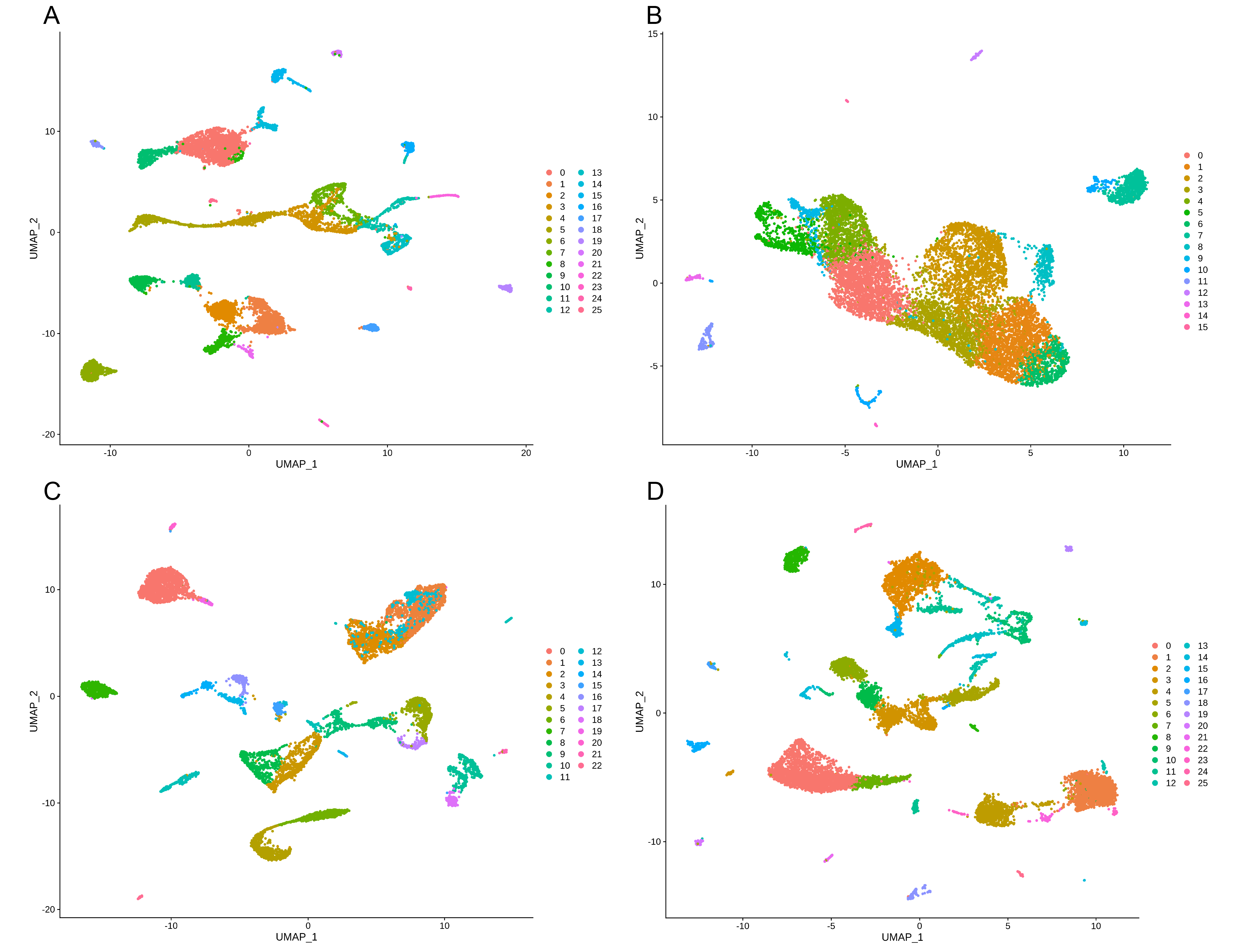

Supplement: Supplementary file 1 — Figure S1. Landscape of four samples. (A) The UMAP showing 9529 cells of N (non‐tumorous) sample including 26 clusters. (B) The UMAP showing 12,078 cells of T1 (tumour) sample including 16 clusters. (C) The UMAP showing 12,107 cells of T2 (tumour) sample including 26 clusters. (D) The UMAP showing 9878 cells of T3 (tumour) sample including 16 clusters. Colour‐coded by their cluster and every dot present a single cell. [file JCMM-29-e70482-s005.tif]

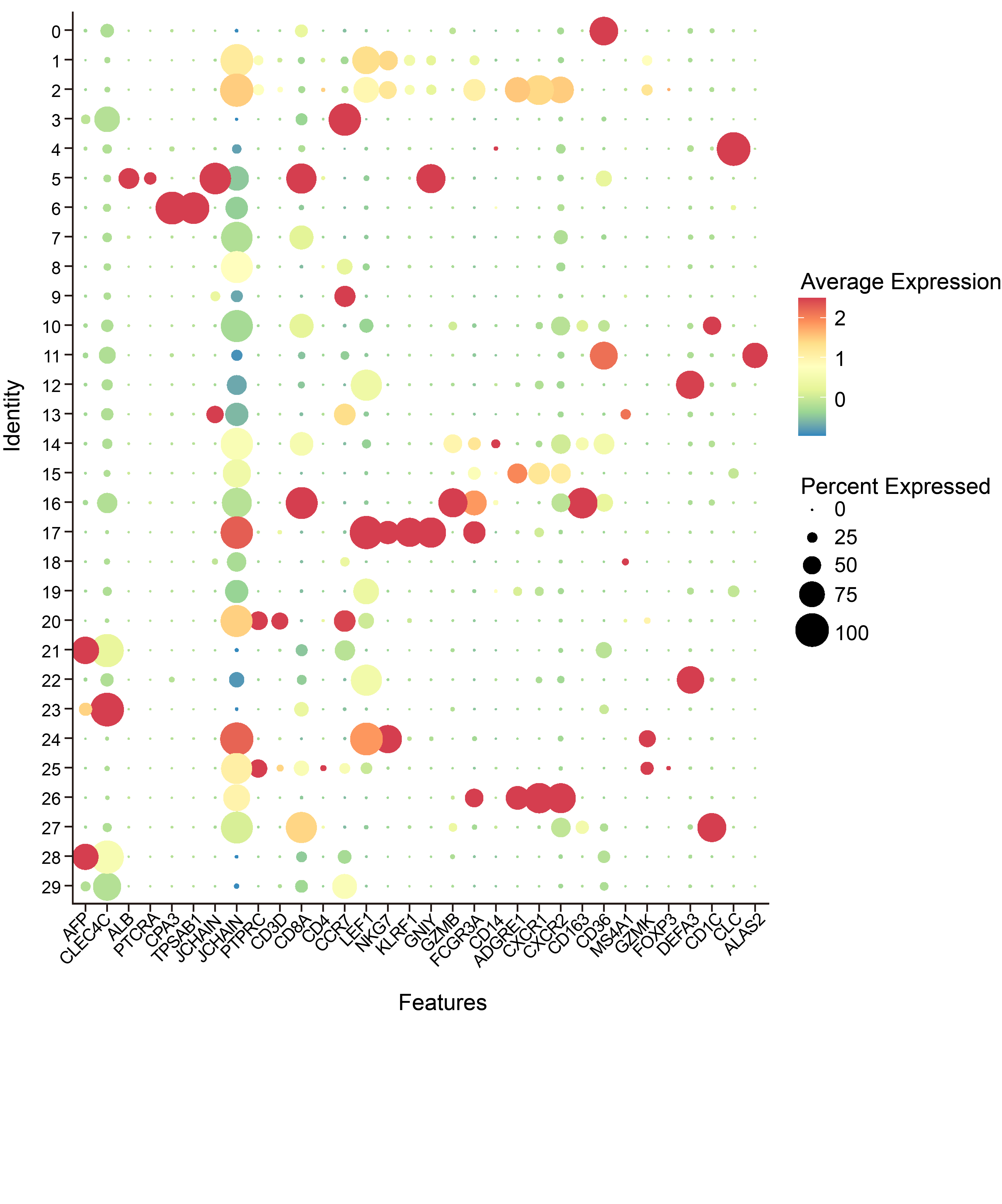

Supplement: Supplementary file 2 — Figure S2. Dot plot showing the most characteristic markers for parenchyma cells each cluster. [file JCMM-29-e70482-s002.tif]

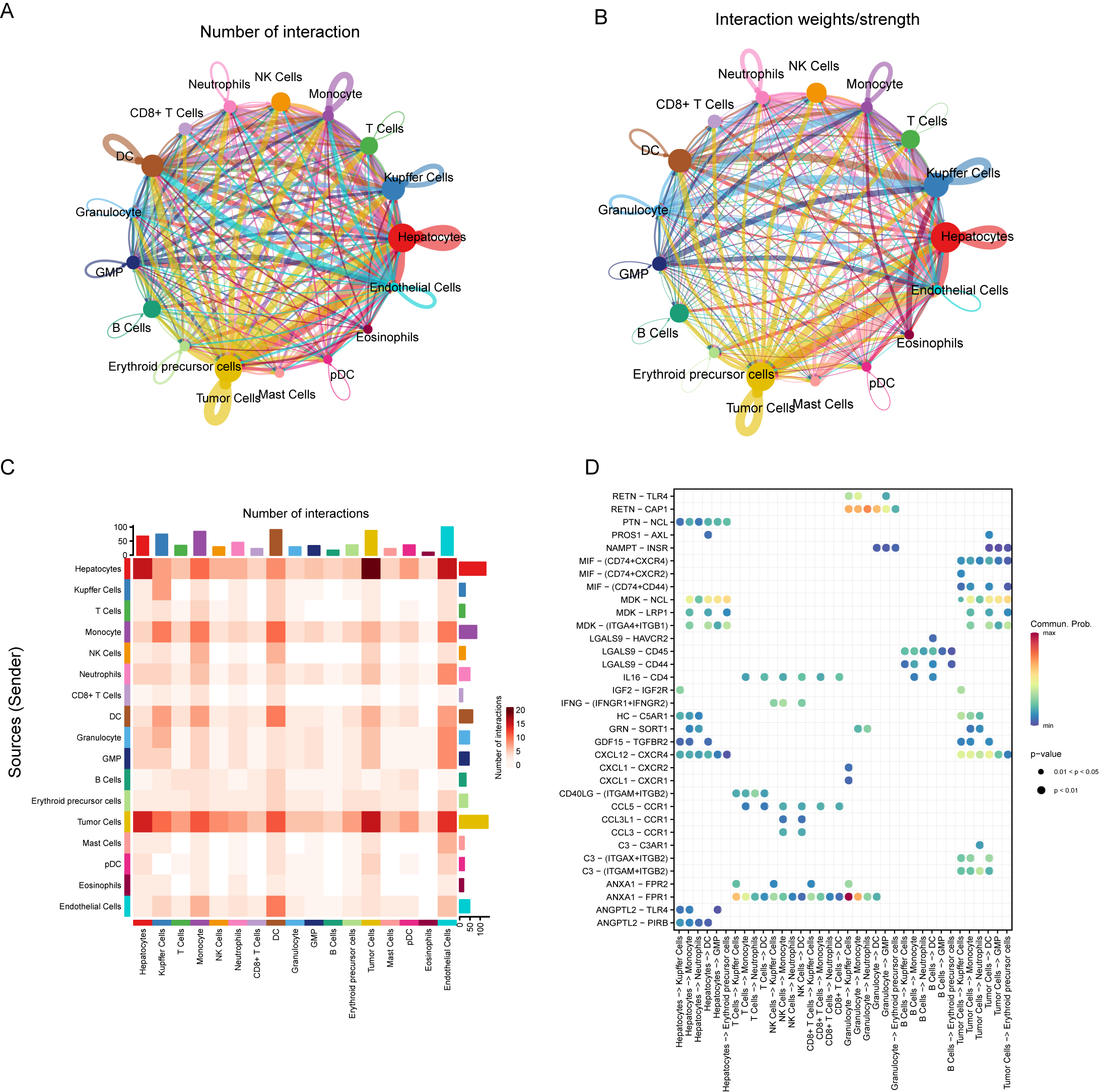

Supplement: Supplementary file 3 — Figure S3. Cell–Cell communication networks in hepatoblastoma. (A) Interaction number among various immune cell types and tumour cells are depicted in the network diagram. (B) Interaction weights and strengths among various immune cell types and tumour cells are depicted in the network diagram. (C) Heatmap depicts the strength of interactions between cell types, with darker colours indicating stronger communication. (D) Statistical significance of the interactions is indicated by p‐values, with thresholds set for p < 0.01 and 0.01 < p < 0.05, providing insights into critical communication pathways within the tumour microenvironment. [file JCMM-29-e70482-s004.tif]

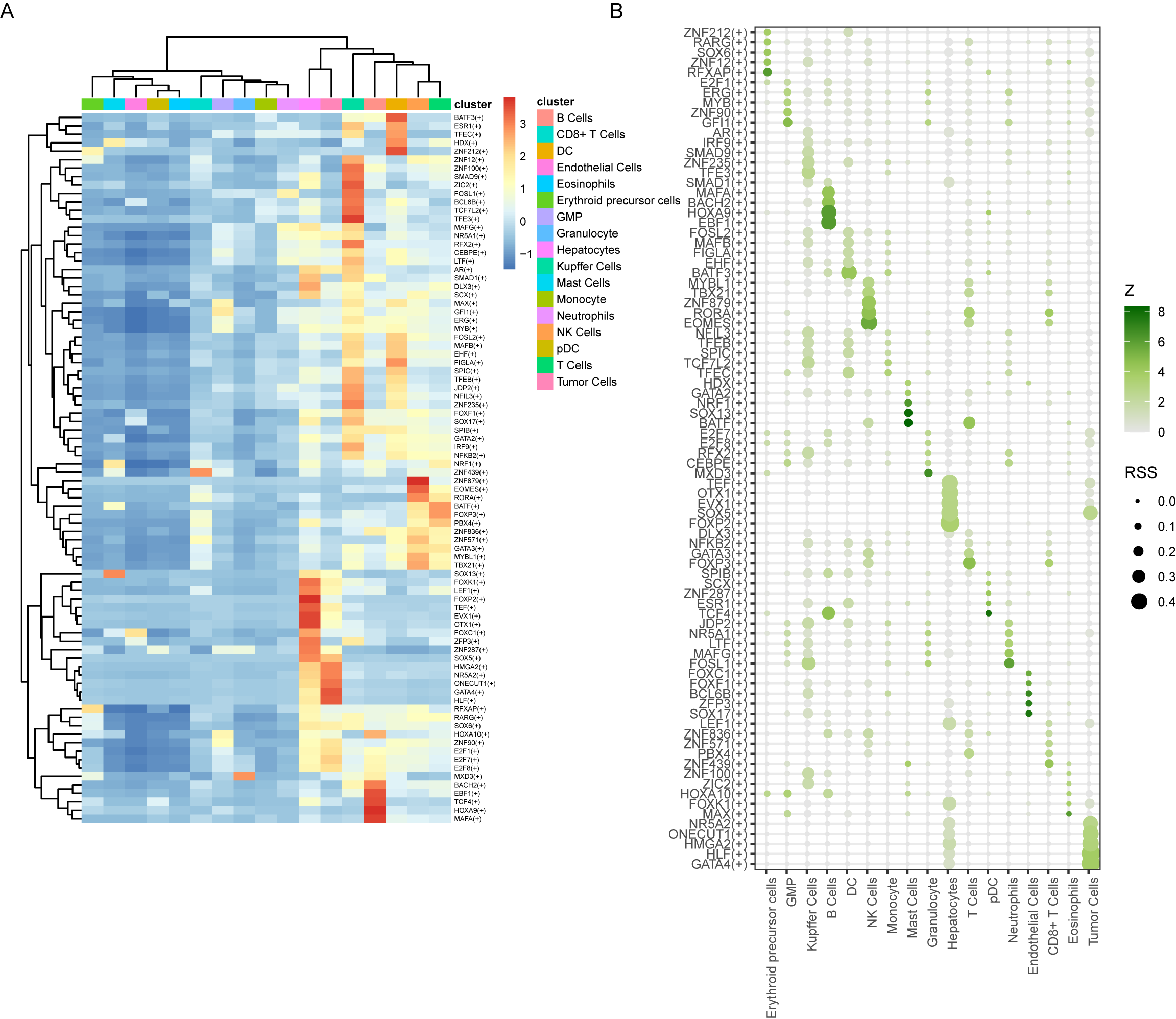

Supplement: Supplementary file 4 — Figure S4. Transcription factor activity in cells of hepatoblastoma. (A) The heatmap displays the activity of key transcription factors (TFs) and their target genes (regulons) in different cell types within the HB tumour microenvironment. (B) Regulon specificity score (RSS): The bar plot shows the regulon specificity scores (RSS) for each cell type, highlighting the most specific regulons for each cell population. [file JCMM-29-e70482-s003.tif]

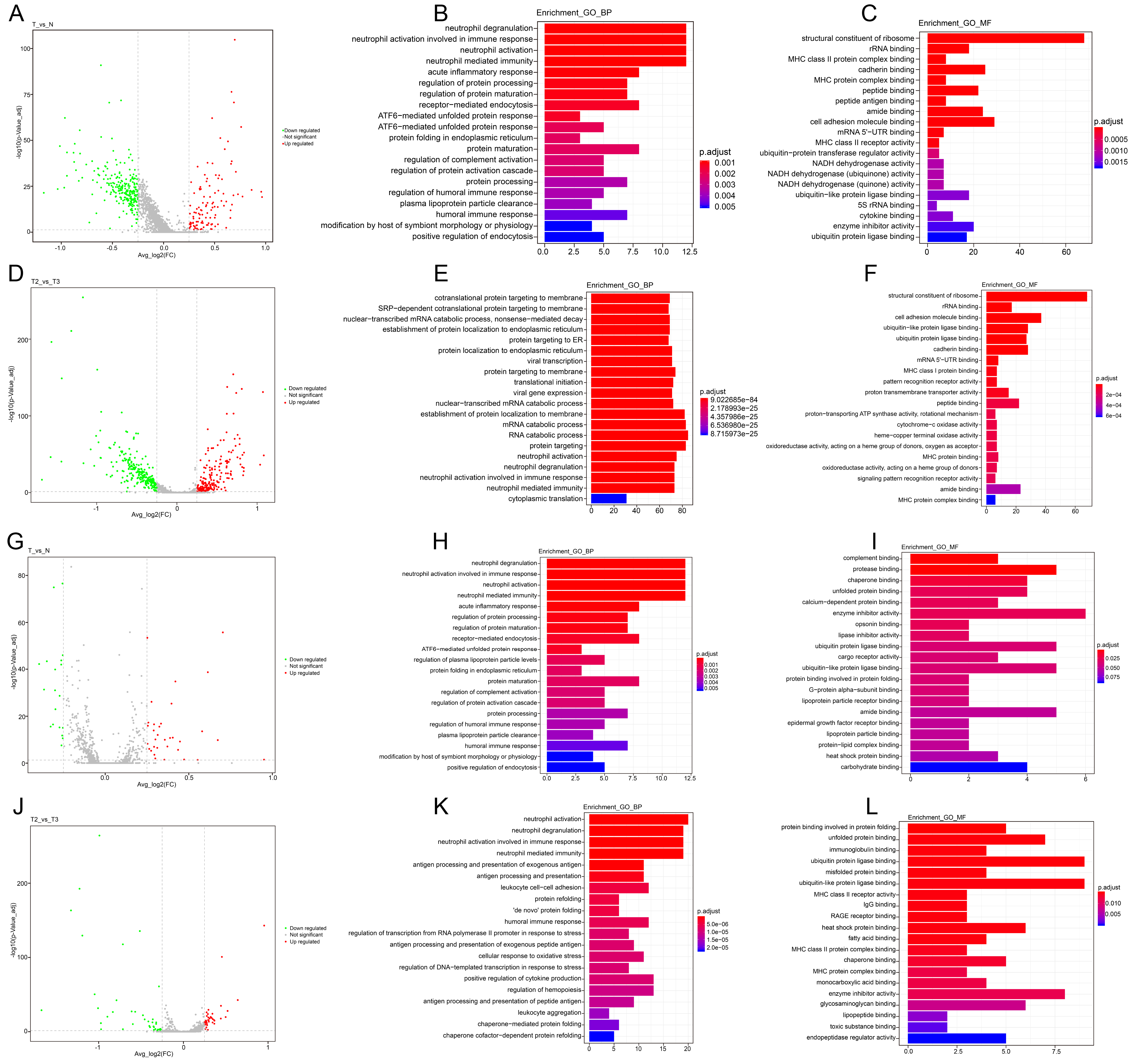

Supplement: Supplementary file 5 — Figure S5. Distinct of myeloid cells in HB tumour tissues. (A) DEGs between T and N in Kupffer cells. (B), (C) The GO analysis of T versus N in Kupffer cells. (D) DEGs between T2 and T3 in Kupffer cells. (E), (F) The GO analysis of T2 versus T3 in Kupffer cells. G, DEGs between T and N in DC. (H), (I) The GO analysis of T versus N in DC. J, DEGs between T2 and T3 in DC. (K), (L) The GO analysis of T2 versus T3 in DC. GO: Gene Ontology; BP: biological process; MF: Molecular Function; DEGs: Differentially expressed genes. [file JCMM-29-e70482-s001.tif]
